# Design and characterisation of a cell exposure system with high magnetic field homogeneity: RILZ coils

**DOI:** 10.3389/fbioe.2024.1337899

**Published:** 2024-03-08

**Authors:** Marco-Xavier Rivera González, Isabel López de Mingo, Alexandra Amuneke Ramírez, Ceferino Maestú Unturbe

**Affiliations:** ^1^ Laboratorio de Bioelectromagnetismo, Centro de Tecnología Biomédica (CTB), Universidad Politécnica de Madrid, Madrid, Spain; ^2^ Escuela Técnica Superior de Ingenieros Informáticos (ETSIINF), Universidad Politécnica de Madrid, Madrid, Spain; ^3^ Escuela Técnica Superior de Ingenieros de Telecomunicación (ETSIT), Universidad Politécnica de Madrid, Madrid, Spain; ^4^ Centro de Investigación Biomédica En Red - Bioingeniería, Biomateriales y Nanomedicina (CIBER-BBN), Centro de Investigación Biomédica en Red, Madrid, Spain

**Keywords:** uniform magnetic field, helmholtz coils, ELF-EMF, intensity homogeneity, EMF exposure system

## Abstract

In vitro studies requiring controlled exposure to low-frequency electromagnetic fields employ exposure systems with different geometries and configurations, the Helmholtz configuration being one of the most widely used. This configuration has limitations in the homogeneity of the spatial distribution of the magnetic field intensity values. We present the design, manufacturing, and characterisation of a new coil system, called RILZ configuration, which improves the distribution of magnetic field intensity values in the three dimensions of space for three different heights in comparison with the traditional circular coils in Helmholtz configuration. In addition, a comparative study of the cellular response in CT2A cultures exposed to a magnetic field of 50 Hz and 100 µT for 48 hrs is performed with both exposure systems. The results of the study show reduced values of deviation from the central value of magnetic field intensity using the RILZ coil system. These differences are statistically significant compared to the Helmholtz configuration for the three Cartesian directions: x (*p* < 0.01), y (*p* < 0.01), z (*p* < 0.01). In addition, the intensity values for three different heights are statistically significantly correlated using the RILZ coil system (*p* < 0.01). The differences in cell behaviour are also statistically significant between the two systems (*p* < 0.01) and may be directly related to the differences found in the distribution of intensity values between the two systems. This study highlights the importance of the homogeneity of the magnetic field intensity generated by the exposure systems used and offers an effective solution to control the magnetic field exposure parameters *in vitro* assays.

## 1 Introduction

In recent years, several *in vitro* studies exposing various cell lines to extremely low-frequency electromagnetic fields (ELF-EMF) (3 Hz–3kHz) are being published in order to understand the mechanisms of interaction between biological systems and this type of non-ionising radiation (Cios et al., 2021; Mansoury et al., 2021; Maleki et al., 2022; Mansoury et al., 2022; Nieminen et al., 2022; Sołek et al., 2022; Elexpuru-Zabaleta et al., 2023; Lazzarini et al., 2023). Magnetic field exposure systems are designed to expose cell samples or devices to controlled magnetic fields and are widely used in scientific and industrial applications (Kirschvink, 1992). These systems can typically contain different coil geometries that act as passive elements for magnetic field generation such as solenoids (Han et al., 2018; Sanie-Jahromi and Saadat, 2018; Costantini et al., 2019; Samiei et al., 2020; Mansoury et al., 2021; Salek et al., 2021; Maleki et al., 2022; Mansoury et al., 2022), circular (Yuan et al., 2018; López et al., 2019; García-minguillán et al., 2020; Xu et al., 2020; Yuan et al., 2020; Asadian et al., 2021) or square (Consales et al., 2018; Sun et al., 2018; Zuo et al., 2020; Elexpuru-Zabaleta et al., 2023; Lazzarini et al., 2023) coils arranged in different configurations, such as the Merrit configuration (Kirschvink, 1992) or the Helmholtz configuration (Kirschvink, 1992; López et al., 2019; García-minguillán et al., 2020; Xu et al., 2020; Asadian et al., 2021; Cios et al., 2021; Lim et al., 2021). The latter is one of the most popular configurations for exposing cell cultures or small animals to ELF-EMF (López et al., 2019; García-minguillán et al., 2020; Xu et al., 2020; Asadian et al., 2021; Cios et al., 2021; Lim et al., 2021). In particular, the use of circular coils arranged in a Helmholtz configuration is one of the most widely used worldwide (Luo et al., 2016; Villarini et al., 2017; Ross et al., 2018; López et al., 2019; Yao et al., 2019; García-minguillán et al., 2020; Asadian et al., 2021; Cios et al., 2021; Lim et al., 2021).

The circular coil system in Helmholtz configuration is composed of two identical coils that are placed in parallel with a distance between their centres equal to the radius of the coils (Helmholtz, 1853). This specific configuration is necessary to achieve a homogeneous ELF-EMF at a central point of the coil system. When current flows in the same direction in both coils, it generates a magnetic field that is uniform in intensity and direction within the spatial line passing through the centre of both coils (Helmholtz, 1853). Its simple design and the uniformity of the magnetic field it produces makes it a widely used configuration in bioelectromagnetics research laboratories (López et al., 2019; García-minguillán et al., 2020; Xu et al., 2020; Asadian et al., 2021; Cios et al., 2021; Lim et al., 2021). However, with space constraints within incubators, Helmholtz coils are limited and do not allow for magnetic field homogeneity over the entire surface of the cell culture plate (Kirschvink, 1992).

The homogeneity of the magnetic field intensity is a crucial parameter in exposure systems used in biological studies, as it plays a significant role in the cellular response of cell cultures; different values of intensity produce different cellular behavioural responses (Misakian et al., 1993; Makinistian et al., 2018). In addition, the introduction of inhomogeneities makes reproducibility of tests impossible (Misakian et al., 1993; Makinistian et al., 2018). Therefore, a non-uniform magnetic field can produce inconsistent results in experimentation. Numerous coil designs have been designed to improve the homogeneity produced by the system in Helmholtz configuration (Kirschvink, 1992).

The main objective of this article is to design, manufacture and characterise a new coil system that improves the homogeneity of the magnetic field in the three dimensions of space compared to the traditional circular coils in Helmholtz configuration and that includes the necessary characteristics for its use *in vitro* studies (biocompatibility, reduced size). For this purpose, the results of the comparison of real magnetic field intensity measurements in three dimensions are presented. In addition, a cellular assay is carried out in which the effect of magnetic field intensity inhomogeneities on the metabolic activity behaviour of a mouse glioblastoma cell culture is evaluated.

## 2 Materials and methods

### 2.1 Simulation in COMSOL multiphysics

**FIGURE 1:**
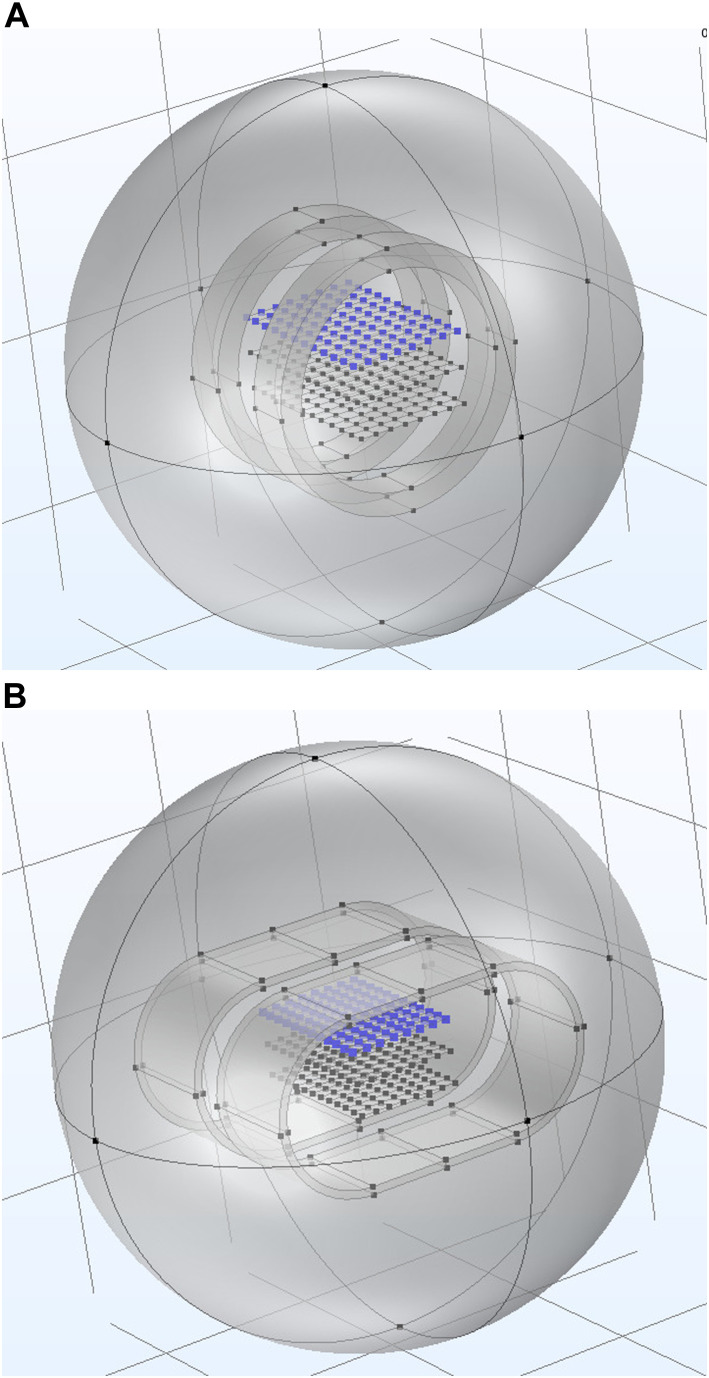
Graphical representation in the COMSOL Multiphysics simulation software of the different coil systems, circular in Helmholtz **(A)** and RILZ **(B)** configuration. The 96 points corresponding to the grid at the three different heights (H1, H0, H-1).

The finite element analysis is performed using COMSOL software (COMSOL Multiphysics^®^ v6.0, COMSOL, Sweden). Firstly, the circular coil system in Helmholtz configuration is simulated. The dimensions, winding material, number of turns and conductor gauge of the circular coils (JEULIN^©^, Évreux, France) available in the bioelectromagnetics laboratory of the Centro de Tecnología Biomédica of the Universidad Politécnica de Madrid are used. These coils have a radius of 6 cm and 96 turns of AWG 18 (1.02 mm) enamelled copper. The COMSOL AC/DC module is used by introducing the coils in a closed air sphere with atmospheric ambient conditions and a temperature of 22°C. The DC current of the model is adjusted until 100 µT is obtained at the centre point between the two coils (0.1 A). In addition, three 96-point arrays are designed with the dimensions of a standard 96-well cell culture plate (126 × 84 × 15 mm), each point being the centre of each of the wells of which the cell culture plate is composed at the height where the culture medium containing the cells is deposited. The three arrays are stacked vertically, with the middle one in the centre of the coils (H0),+/-1.7 cm apart (H1, H-1), mimicking the arrangement of three 96-well cell culture plates one on top of the other. The COMSOL representation of the model can be seen in Figure 1A. In the model calculation, the magnetic field intensity values are obtained at the 96 points of each of the arrays at the three different heights (H1,H0,H-1) and in the three Cartesian directions (x,y,z).

Secondly, the simulation of the proposed new coil system is carried out. During the design, different requirements were taken into account: i) the material used had to be the same as the one used in the previous simulation, ii) the dimensions of the coils had to be the minimum that would improve the homogeneity of the distribution of the intensity values with respect to the traditional system to facilitate their manipulation during the *in vitro* assays, iii) the main component of the magnetic field should be located in a single direction parallel to the surface on which the cells are arranged on the cell culture plate, considering the values of the two remaining components as close to 0 as possible. This new selected configuration, hereinafter referred to as “RILZ coils”, consists of two coils each in the form of a capsule consisting of two semicircles with a radius of 5 cm joined by two 10 cm long straight lines. The width for winding is 7 cm and the distance between the coils is 3.5 cm. The RILZ coils are simulated under the same conditions as the traditional system above, embedded in a closed sphere with atmospheric ambient conditions and a temperature of 22°C. They have 222 turns of enamelled copper AWG 18. The coils are stimulated in DC with a current of 0.05 A to obtain 100 μT at the centre point between the two coils. As in the simulation of the circular coil system, three arrays of 96 points at three different heights located in the middle of the coils are introduced, obtaining the magnetic field intensity values for each of the points in the three Cartesian directions (x,y,z) and each of the heights (H1,H0,H-1) in the model calculation. The COMSOL representation of the model can be seen in Figure 1B.

After obtaining the magnetic field intensity values at the 96 points of the matrix and at the three different heights for each of the systems, MATLAB R2022b software (The MathWorks^©^, United States) is used to process and visualise the 3D plots of the distribution of the magnetic field intensity values.

### 2.2 Design and manufacture of coils

**FIGURE 2:**
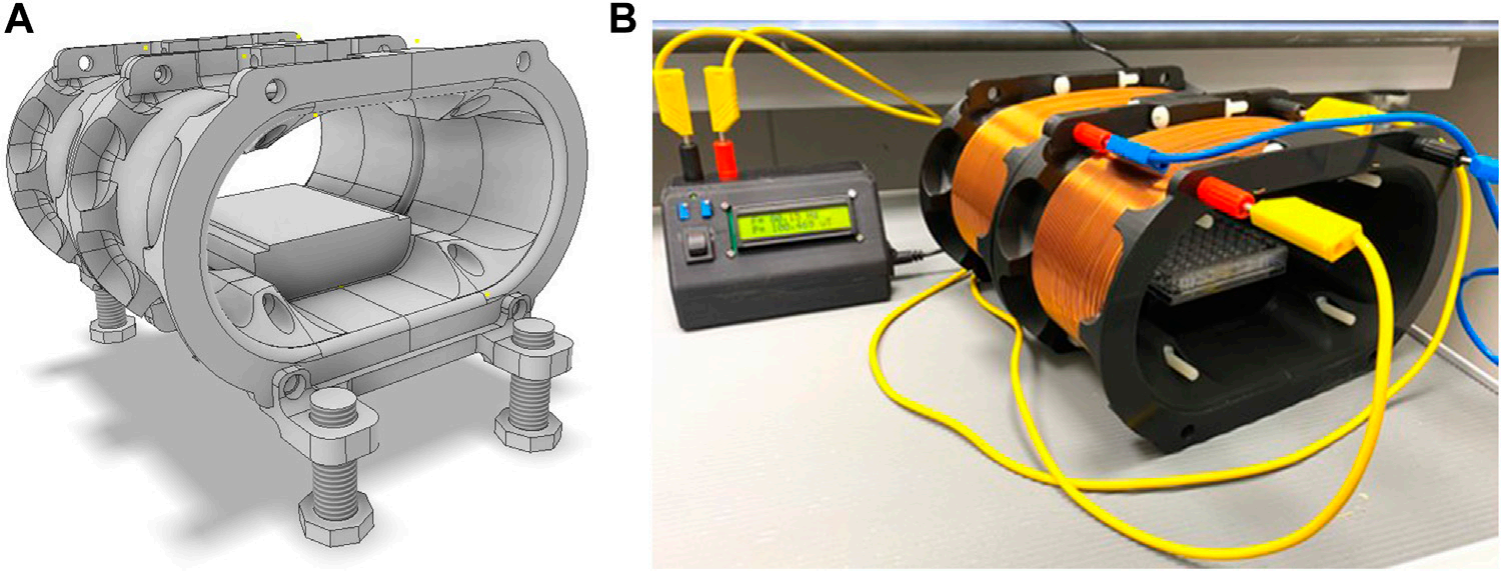
**(A)** Graphical representation of the RILZ coil skeleton using Autodesk Inventor software. **(B)** Final result of the manufactured RILZ coil system connected to the power electronics.

The skeleton of the RILZ coils is designed using Autodesk Inventor Professional software (Autodesk^®^ Inventor^®^, v2023, California, United States) (Figure 2A). For the 3D printing of the coils, UltiMaker Cura software (UltiMaker^©^, v5.2.2, Netherlands) is used. The IIIP Monoprice Delta Pro 3D Printer (Monoprice, California, United States) and the plastic material polylactic acid (PLA) (3D Printer Filament, ANYCUBIC-US, cat. no. HPLKK-103) are used. Each coil is printed in four different parts which are held together by nylon screws (Caterpillar Red, cat. no. 0727040189389) to avoid magnetic field disturbances that can be produced by metals in the coil structure. Each of the coils has 222 turns of AWG 18 gauge enamelled copper (Cetronic, cat. no. 0727040189389). In addition, the structure includes a height adjustment system to avoid interference generated by the metal trays of the incubators (Figure 2B).

### 2.3 Description of the electronic system used for supplying power to the coils

**FIGURE 3:**
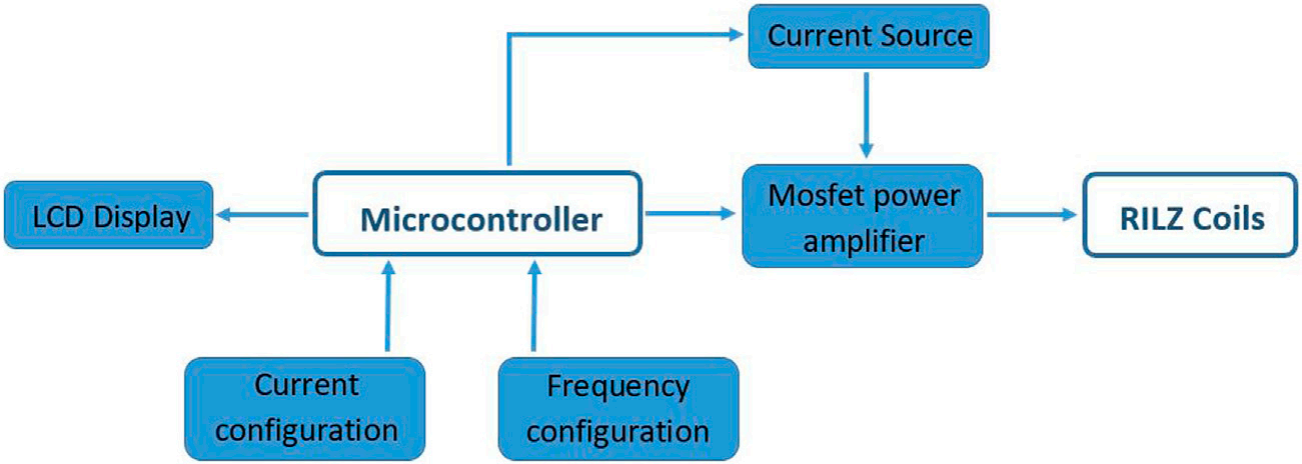
Block diagram of the electronic system designed to power the RILZ coils.

The electronic system responsible for powering both sets of coils for magnetic field generation has been designed and developed at the Biomedical Technology Centre. The system generates a square signal controlled by a microprocessor. The values of frequency and current intensity are displayed on an LCD screen and are configured using precision potentiometers connected to the microcontroller’s analogue-to-digital converter (ADC). Frequency is set between DC and 200 Hz, using interrupts generated by the microcontroller’s internal timer. The current intensity is set between 0 and 2 Amps, using a current amplifier based on MOSFET-type power transistors controlled by the microcontroller. The output of this current amplifier feeds the RILZ coils. This circuit will be responsible for powering both coil systems during the development of the cell cultures. Figure 3 shows the block diagram of the designed circuit.

**FIGURE 4:**
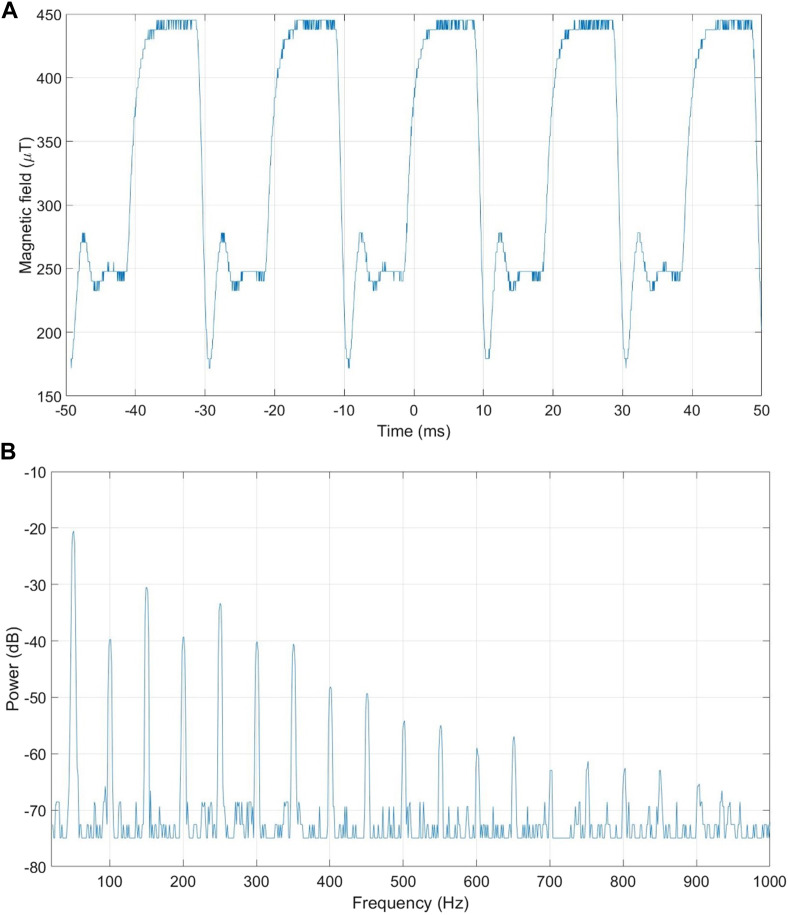
Signal recorded in the RILZ coils. **(A)** time domain, **(B)** frequency domain.

Real-time magnetic field intensity measurements are taken to document the waveform, intensity, frequency and harmonics of the signal generated by the electronics and the RILZ coils. Measurements are performed with a LakeShore Model 480 Fluxmeter (Lake Shore Cryotronics^©^, Ohio, United States) with a triaxial probe model MMZ-2502-UH (Lake Shore, Cryotronics^©^, Ohio, United States). The analogue output of the Fluxmeter is used and connected to an oscilloscope model TDS 2024C (Tektronix^©^, Oregon, United States). The resolution of the Fluxmeter is 1 μT/mV; and it has a signal offset of 250 mV. The recorded signal is processed on a computer with MATLAB R2022b software (The MathWorks^©^, United States). Figure 4A shows the recorded signal with an RMS value of 351.30 µT; after removing the equipment offset (250 µT) the RMS value of the signal is 101.30 µT. Figure 4B shows the frequency response of the recorded signal, with a fundamental frequency of 50 Hz and its corresponding harmonics in dB.

### 2.4 Measurements of magnetic field intensity

**FIGURE 5:**
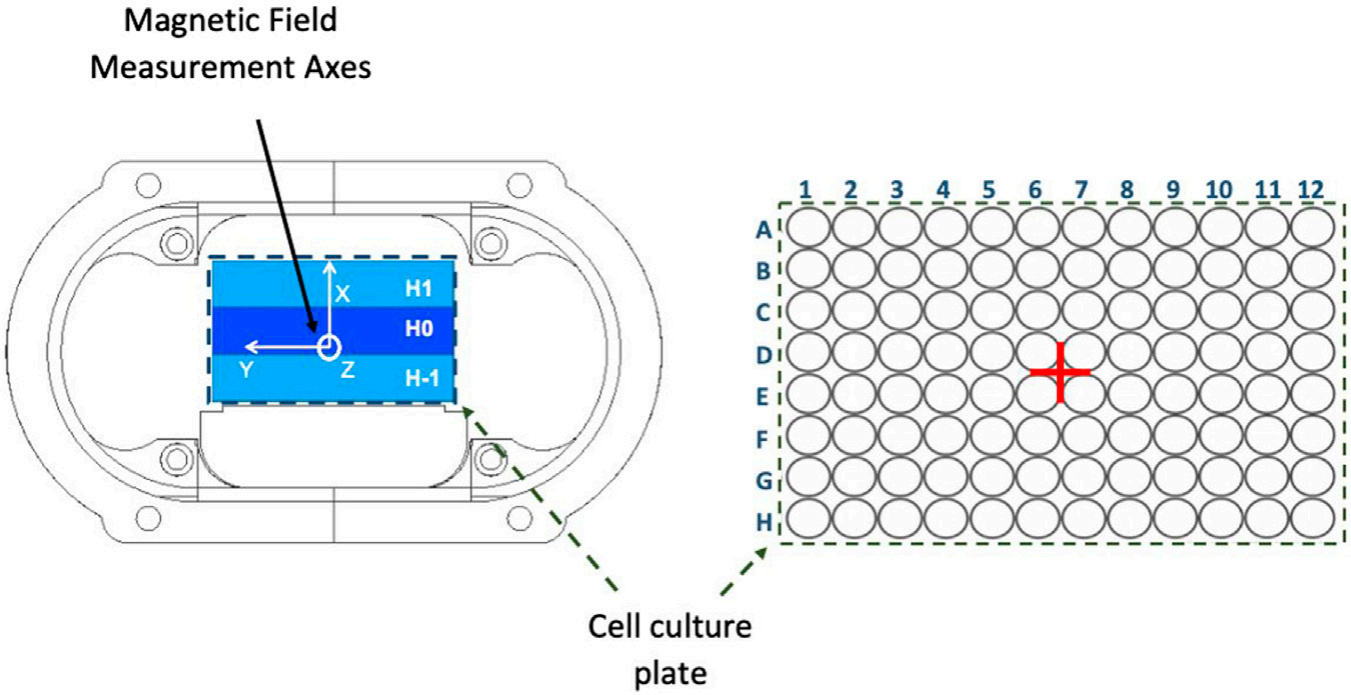
Graphical representation of the different components of the measured magnetic field vector (x, y, z) and the different measuring heights (H-1, H0, H1). (+) Central measuring point.

Magnetic field intensity measurements are taken using a LakeShore Model 480 Fluxmeter (Lake Shore Cryotronics^©^, Ohio, United States) with a Model MMZ-2502-UH triaxial probe (Lake Shore, Cryotronics^©^, Ohio, United States). Each of the coil systems, connected in series, is DC powered through an AIM-TTI Instruments (Thurlby Thandar Instruments, UK) model QL355TP power supply limited in current to supply the 100µT magnetic field intensity value at the centre point of the coils (circular coils in Helmholtz configuration, I = 0.064 A; RILZ coils, I = 0.045 A) (Figure 5). Both the central measurement and the rest of the measurements are calculated as follows, $P\;\left(µT\right)=P\left[ON\right]\left(µT\right)-P\left[OFF\right]\left(µT\right)$ , where *P* is a given measurement point; *P[ON]* is the magnetic field intensity value measured with the generator switched on and y *P[OFF]* is the magnetic field intensity value measured with the generator switched off.

A total of 291 intensity measurements (resulting from the subtraction of on/off device intensities) are made per system, 288 corresponding to the positions of each of the points of the grid used for the calculation of the simulation model in COMSOL, as indicated in Section 2.1. Simulation in COMSOL Multiphysics, and 3 corresponding to the central point at the three different heights (H-1, H0 and H1). The power supply current is set to the value that delivers 100 µT of magnetic field intensity at the centre point of each cell culture plate (Figure 5).

### 2.5 Cellular line

CT2A mouse glioblastoma cells were obtained from the Instituto Cajal de Madrid of the Consejo Superior de Investigaciones Científicas (CSIC), Spain, and were maintained in monolayer culture in Dulbecco’s Modified Eagle’s Medium with Elevated Glucose (DMEM) (DDBiolab, w/L-Glutamine, without sodium pyruvate, cat. no. L0102-500), supplemented with 10% fetal bovine serum (DDBiolab, cat. no. P30-3302), 1% L-Glutamine (DDBiolab, 200 mM, cat. no. P04-80100) and 1% penicillin/streptomycin (DDBiolab, penicillin 5,000 Ul/mL, streptomycin 5). Cells were cultured at 37°C under an atmosphere of 5% CO2 in air. Cell subpopulations were prepared by cell passaging twice a week when the cell culture plates were close to 90% confluence.

### 2.6 Exposure conditions

The cells were seeded in two 96-well cell culture plates at a density of 80,000 cells/mL. Each of the cell culture plates was exposed simultaneously in the two exposure systems used, on the one hand, the Helmholtz circular coil system, on the other hand, the RILZ coil system, powered by the electronic system described in Section 2.2. Description of the electronic system, used for supplying power to the coils. Both systems were placed in two identical Thermo Scientific 3111 series II incubators (Thermo Fisher Scientific Inc., Massachusetts, United States). The position of the cell culture plates corresponded to the H0 height in both systems. Both sets of coils were raised 5 cm in supports with respect to the metal tray of the incubator, so they were not in direct contact with it in order to eliminate the noise generated by the induction of the magnetic field by direct contact with the grounded metal surface (Figure 2A). The magnetic stimulation field had an intensity of 100 µT (exposure limit set by recommendation 1999/519/EC in Spain for a frequency of 50 Hz) (Consejo de la Unión Europea, 1999), a frequency of 50 Hz (corresponding to the frequency of alternating electrical current in Europe) using a train of square pulses as waveform as indicated in 2.2. Description of the electronic system. Exposure was uninterrupted for 72 h from seeding. Magnetic field intensity measurements were performed with the stimulation equipment off in both incubators using the LakeShore Model 480 Fluxmeter (Lake Shore Cryotronics^©^, Ohio, United States) and the triaxial probe model MMZ-2502-UH (Lake Shore Cryotronics^©^, Ohio, United States) with the absolute value of intensity recorded in the three directions of space, x (23.14 ± 0.39 µT), y (42.30 ± 1.19 µT), z (5.96 ± 0.49 µT). Three replicates were performed in three independent experiments for each of the exposure systems used, the exposure being simultaneous.

### 2.7 Metabolic activity test

After the exposure time, cell metabolic activity was assessed using the 3-(4,5-dimethylthiazol-2-yl)-2,5-diphenyl-2H-tetrazolium bromide (MTT) assay (Biotium, MTT Cell Viability Assay Kit, cat. no. 30006). The assay was performed according to the manufacturer’s instructions. Briefly, 10 µL of MTT agent was added to each well of the 96-well cell culture plate. The cell culture plates were then incubated in the dark for 4 h. After this period, 200 µL of dimethyl sulfoxide (DMSO, Corning Media Tech, Cat. no. 15303671) was added to each of the wells and resuspended to break up the crystals formed.

The absorbance was measured using a HEALES model MB-580 microplate reader (HEALES, Shenzhen, China) at wavelengths of 570 nm and 630 nm (corresponding to the background signal).

The deviation from the mean of the absorbance values obtained for each of the exposure configurations in each of the wells of the cell culture plate is calculated. The representation of the heat maps for the standard deviation values obtained with respect to the mean absorbance is performed with Prism 9 software (GraphPad^©^ Software, v.9.3.1., Massachusetts, United States).

### 2.8 Statistical analysis

The statistical analysis of the results is carried out using SPSS Statistics software (IBM SPSS Statistics^©^ Software, v.29.0.0.0., New York, United States). For the descriptive statistics section, the mean values (± standard deviation, SD) and the maximum and minimum values are used. The absolute values of the deviation from the reference value taken in the measurements are used:

(i) X-axis (reference value: 0 µT): $D_{x}\;\left(µT\right)=\left|P_{x}\left(µT\right)-0\;µT\right|$

(ii) Y-axis (reference value: 0 µT): $D_{y}\;\left(µT\right)=\left|P_{y}\left(µT\right)-0\;µT\right|$

(iii) Z-axis (reference value: 100 µT): $D_{z}\;\left(µT\right)=\left|P_{z}\left(µT\right)-100\;µT\right|$

Where *D* is a point of deviation from the reference value determined with directional components $D_{x}$ (X-axis), $D_{y}$ (Y-axis), $D_{z}$ (Z-axis); *P* is a measuring point determined with directional components $P_{x}$ (X-axis), $P_{y}$ (Y-axis), $P_{z}$ (Z-axis).

The absorbance values are expressed in the same way taking as reference value the average absorbance of the evaluated wells.

**FIGURE 6:**
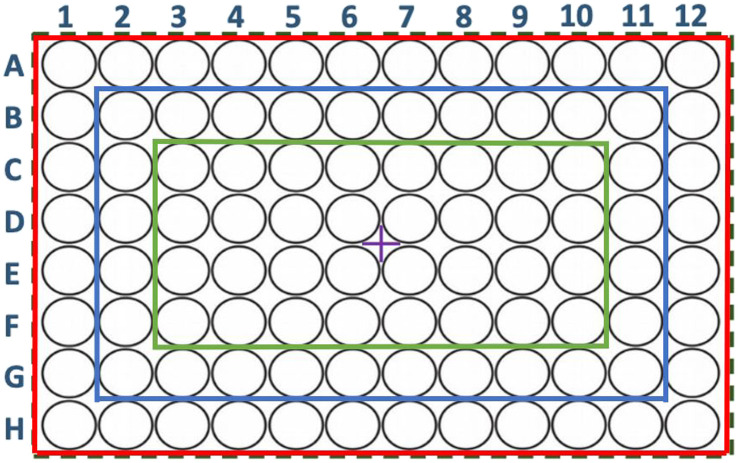
Representation of a 96-well cell culture plate. In red the bounded enclosure containing 96 wells (i), in blue the bounded enclosure containing 60 wells (ii) and in green the bounded enclosure containing 32 wells (iii). (+) Central measuring point.

To perform the comparative analysis between the variables, the 96-well cell culture plate is sectioned from the wells furthest from the centre of the cell culture plate towards the part closest to the central reference point as follows (Figure 6):

(i) All 96 wells of the cell culture plate are used. Total number of wells: 96.
(ii) Columns 1 and 12 and rows A and H are removed. Total number of wells: 60.
(iii) In addition to columns 1 and 12 and rows A and H (ii), columns 2 and 11, and rows B and G are deleted.

Several comparative analyses are carried out between the variables. In all of them, the normality of the data is assessed according to the Kolmogorov-Smirov statistical test (samples greater than 50) or the Shapiro-Wilk statistical test (samples less than 50) with a 95% CI. If the samples used have a normal distribution, the quantitative variables are compared using the ANOVA statistical test of analysis of variance with a 95% CI. Otherwise, the statistic applied is the U-Mann Whitney test with a 95% CI. The statistical correlation of quantitative variables is analysed using the Pearson correlation (for normally distributed data) or the Spearman correlation (for non-normally distributed data) with a 95% CI.

## 3 Results

### 3.1 Results of simulation

The main objective of this section is to compare the distribution in the three Cartesian directions of space (x,y,z) and at three different heights (H1, H0, H-1) of the magnetic field intensity values obtained after simulating the circular coil system in Helmholtz configuration and the RILZ coil system. For this purpose, after simulating the two systems, the intensity values of each of the points of a 96-point grid representing the arrangement of the wells in a 96-well cell culture plate are obtained. The metrics used are calculated from the deviation values with respect to the reference values for each of the axes as indicated in Section 2.8.

#### 3.1.1 Middle height, H0

**FIGURE 7:**
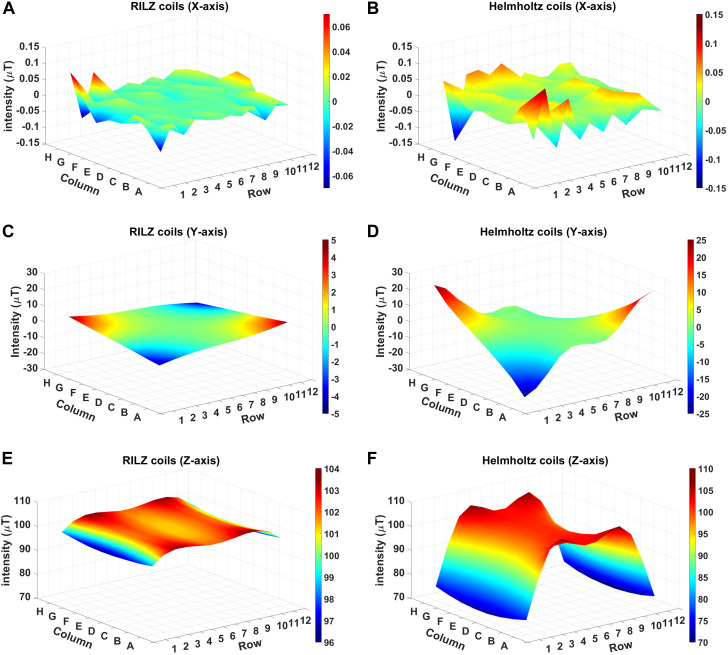
3D graphical representation of the simulated intensity distribution in the different exposure systems, RILZ **(A,C,E)** and Helmholtz **(B,D,F)** evaluated at 96 points, in the different Cartesian directions, x **(A,B)**, y **(C,D)**, z **(E,F)** when supplied with DC power with the centre point of the grid at 100 µT.

Figure 7 shows the distribution of simulated magnetic field intensity values in the three directions of x, y, and z space for the different coil systems evaluated, RILZ and circular in Helmholtz configuration. Qualitatively, the reduced variability of the intensity data of the RILZ coil system compared to the Helmholtz circular coil system can be seen, the former having flat figures of the intensity distribution in volume compared to the traditional exposure system.

The average, minimum and maximum values of the intensity deviation for the two coil systems evaluated in the three Cartesian directions can be seen in Table 1. The average values obtained indicate that in all three directions (x,y,z), the RILZ configuration achieves deviation values that are at least half of those obtained by the Helmholtz configuration. This better distribution of values is confirmed by the minimum and maximum values, with the maximum deviation values being closer to 0 in the RILZ configuration than in the Helmholtz configuration. In addition, a smaller range between the maximum and minimum value is found in the RILZ configuration with values not exceeding 5 µT difference in any of the three Cartesian directions.

**TABLE 1:** Descriptive statistics of the deviation values with respect to the reference value for the three Cartesian axes (x, y, z), of both exposure configurations, RILZ–HELMHOLTZ obtained in the simulation in COMSOL Multiphysics for the middle height, H0.

Coils system	Deviation	Average (±SD) [µT]	Minimum value [µT]	Maximum value [µT]
RILZ	$D_{x}$	0.012 (±0.015)	0.00	0.08
	$D_{y}$	1.16 (±1.18)	0.03	4.66
	$D_{z}$	2.06 (±0.87)	0.01	3.55
Helmholtz	$D_{x}$	0.022 (±0.025)	0.00	0.14
	$D_{y}$	5.96 (±6.77)	0.09	24.48
	$D_{z}$	8.90 (±9.34)	0.20	28.90

The descriptive statistics values of the magnetic field intensity distribution for both systems show that the RILZ coil system produces a more homogeneous magnetic field over the whole 96 points of the model evaluation grid for a height H0.

#### 3.1.2 Heights H1 and H-1

**FIGURE 8:**
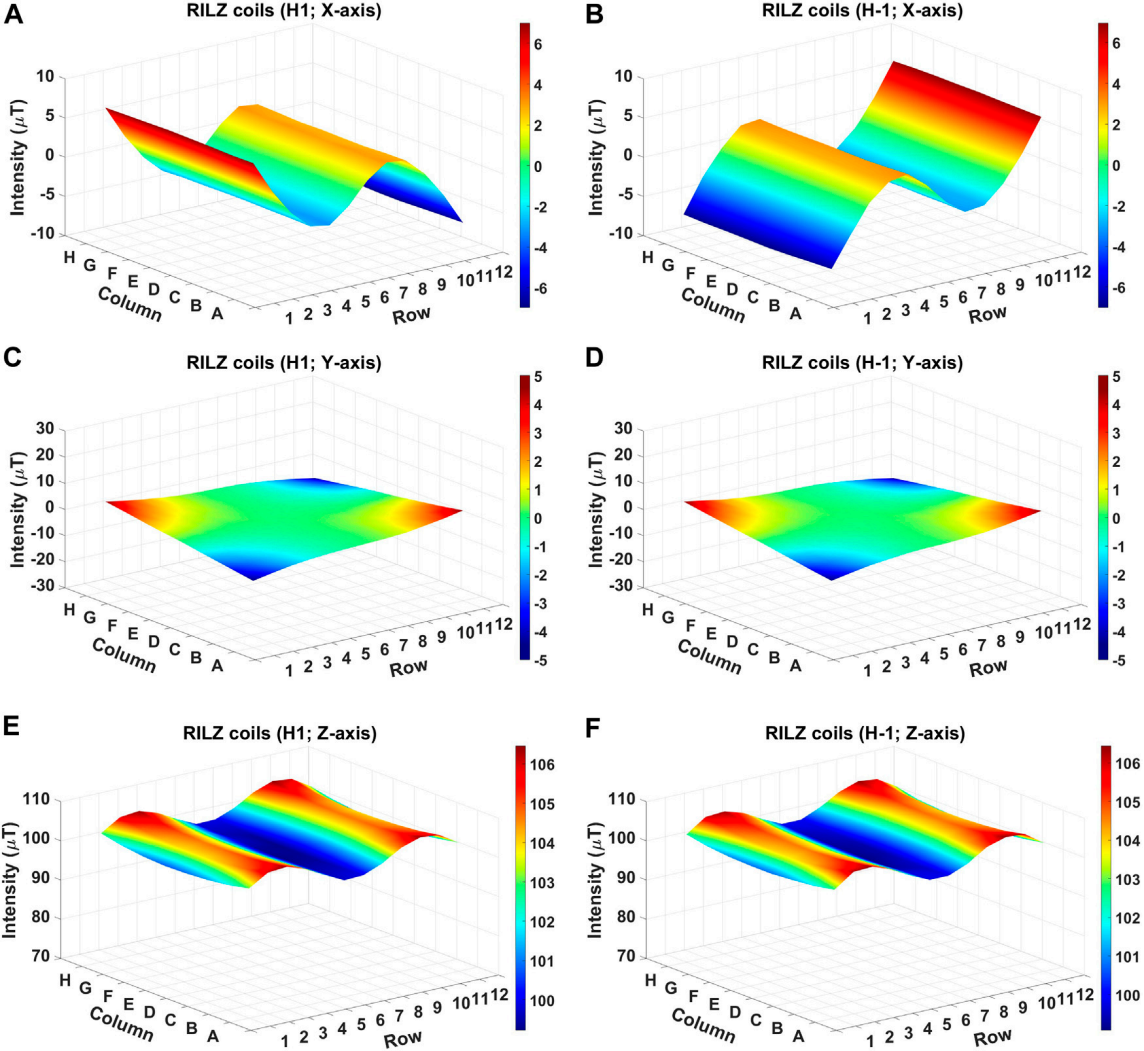
3D graphical representation of the simulated intensity distribution in the different heights H1 **(A,C,E)** and H-1 **(B,D,F)** of the RILZ coils system evaluated at 96 points, in the different Cartesian directions, x **(A,B)**, y **(C,D)**, z **(E,F)** when supplied with DC power with the centre point of the grid at 100 µT.

Figure 8 shows the distribution of simulated magnetic field intensity values in the three directions of x, y, and z for the RILZ coil system at two different heights H1 and H-1. The similarity of the data can be seen not only for the upper height *versus* the lower height (H1 vs. H-1), but also for the lower height *versus* the middle height (H1 vs. H0; H-1 vs. H0) (Figures 8A, C, E; Figure 7).

Table 2 shows the average, maximum and minimum values obtained with the RILZ coil system in the three Cartesian directions (x,y,z) at the three different heights tested (H1,H0,H-1). The average deviation values show to be identical at heights H1 and H-1, with a difference of less than 1 µT in all directions with respect to the middle height H0 (Table 1), except in the X-axis where the difference between heights is 2.9 µT. These data allow us to evaluate the use of three-point grids in taking real intensity measurements with the RILZ system at three different heights while preserving as much magnetic field homogeneity as possible.

**TABLE 2:** Descriptive statistics of the deviation values with respect to the reference value for the three Cartesian axes (x,y,z) of RILZ configuration obtained in the simulation in COMSOL Multiphysics for the three different heights (H1,H0,H-1).

Height	Deviation	Direction	Average (±SD) [µT]	Minimum value [µT]	Maximum value [µT]	Range (maximum value–minimum value) [µT]
H1	$D_{x}$	X	2.90 (±2.00)	0.38	6.99	6.61
	$D_{y}$	Y	1.10 (±1.16)	0.02	4.59	4.57
	$D_{z}$	Z	2.96 (±1.94)	0.06	6.45	6.39
H-1	$D_{x}$	X	2.90 (±2.01)	0.41	7.00	6.59
	$D_{y}$	Y	1.10 (±1.15)	0.02	4.48	4.46
	$D_{z}$	Z	2.96 (±1.94)	0.14	6.43	6.29

The simulated values of deviation from the average intensity distribution in the three Cartesian directions obtained in the comparison between the two exposure systems and the evaluation of the distribution of magnetic field intensity at three different heights of the RILZ coil system confirm the hypothesis of a better magnetic field distribution of the new system, which allows to continue taking real measurements.

### 3.2 Results of field intensity measurements

This section presents the results obtained after taking real magnetic field intensity measurements for three 96-well plates stacked in the centre of two different coil systems, the traditional Helmholtz circular coil system and the RILZ coil system. The metrics used are calculated from the deviation values from the reference values for each of the axes as indicated in section 2.8. Statistical Analysis.

#### 3.2.1 Middle height, H0

**FIGURE 9:**
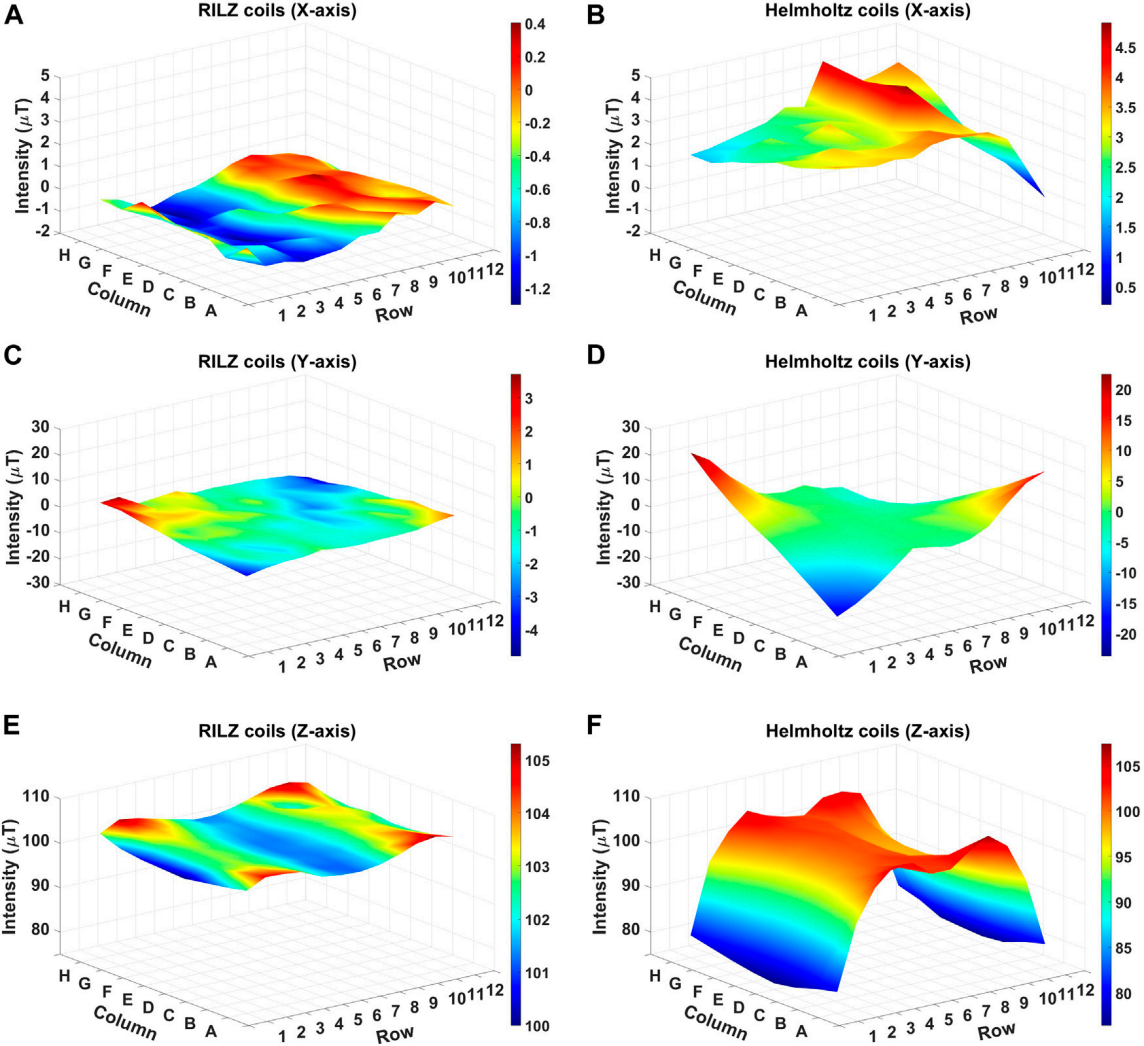
3D graphical representation of the intensity distribution of the measurements in the different heights H1 **(A,C,E)** and H-1 **(B,D,F)** of the RILZ coils system evaluated at 96 points, in the different Cartesian directions, x **(A,B)**, y **(C,D)**, z **(E,F)** when supplied with DC power with the centre point of the grid at 100 µT.

Figure 9 shows the three-dimensional distribution of the intensity values for each of the systems in the three Cartesian directions. Qualitatively, it can be seen that in the three axes (x,y,z) the distribution of the intensity values in the RILZ system in the 96 points measured is more homogeneous than that obtained in the Helmholtz circular coil system, the graphs of this first system having a flatter shape with respect to the second. In numerical data, evaluating the deviations, in the main direction of the application of the magnetic field of 100 µT (Z axis), the RILZ system obtains average deviation values of less than 3 µT for all the different sets of wells evaluated and a maximum value of 5.30 µT, while the Helmholtz system exceeds 7 µT if the number of wells evaluated is 96, with a maximum value of 23.60 µT. In this system, the average deviation values decrease as the number of wells tested decreases, with an average value of 4.4 µT and 2.60 µT if the number of wells is 60 and 32, respectively. This indicates that in the Helmholtz exposure system the increase in deflection is directly proportional to the size of the tested surface on the cell culture plate, as it moves away from the centre of application of the magnetic field. On the X-axis, the Helmholtz coil system again obtains higher values than those found in the RILZ system, with deviations that are double those obtained with the new system. Even greater is the difference found in the intensity deviation values between both systems in the Y axis, with the average deviation values of the Helmholtz system being higher than 5.0 µT when the 96 wells are evaluated, with a maximum value of 23.7 µT, and decreasing to 3 μT and 2 µT when the evaluated wells are 60 and 32 wells, respectively. In this case, the deviation values of the RILZ system do not exceed 1.5 µT in all the wells evaluated, with a maximum value of 4.8 µT. The descriptive statistics values can be seen in Figure 9 and can be found in Supplementary Table S1.

**FIGURE 10:**
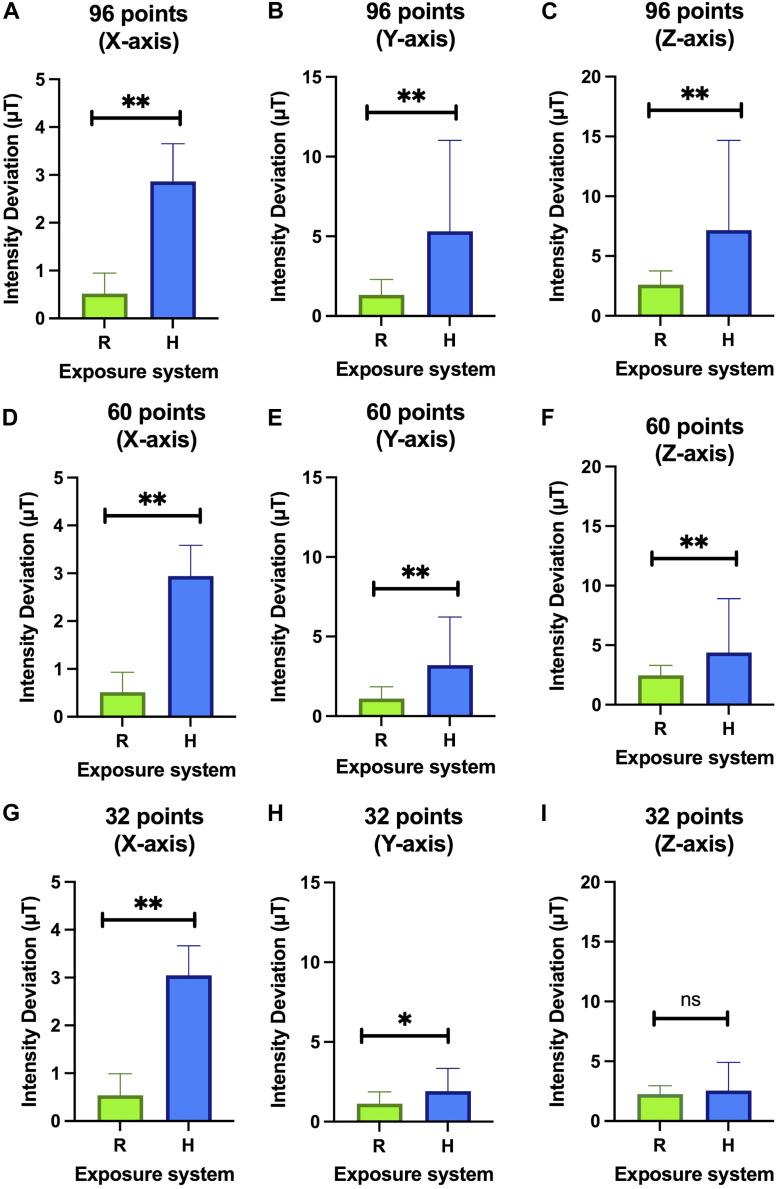
Bar chart representing the average value and the standard deviation of the deviation values from the reference value for each of the systems (R: RILZ; H: Helmholtz). The three Cartesian components, x **(A,D,G)**, y **(B,E,H)**, z **(C,F,I)** are evaluated at 96 points **(A,B,C)**, 60 points **(D,E,F)** and 32 points **(G,H,I)**. The ANOVA statistical test is applied with a 95% confidence interval. (n.s.) non-significant. (*) *p*-value < 0.05; (**) *p*-value < 0.01.

Statistically significant differences in the magnetic field intensity distribution between the two systems are found when 96 points are evaluated for the three Cartesian directions, x (*p* < 0.001), y (*p* < 0.001) and z (*p* < 0.001), 60 points also in the three Cartesian directions, x (*p* < 0.001), y (*p* < 0.001) and z (*p* = 0.001). 001) and z (*p* < 0.001), 60 points also in the three Cartesian directions, x (*p* < 0.001), y (*p* < 0.001) and z (*p* = 0.002) and at 32 points in the Cartesian directions x (*p* < 0.001) and y (*p* = 0.037), but not at z (*p* = 0.474) (Figure 10). These results, taken together with Figure 8 and the descriptive statistics data (Figure 9), show that the differences between the two systems are evident for the *X* and *Y* axes with the RILZ system having values close to 0 on these axes and a more homogeneous distribution. On the Z-axis, the main axis of the magnetic field application, these results indicate that in the evaluation of 96 and 60 wells the RILZ system obtains statistically significant results in the distribution of intensity values being more homogeneous. On the other hand, if the number of wells evaluated is 32, both systems show a homogeneous and not statistically significant distribution of intensity values.

#### 3.2.2 Heights H1 and H-1

**FIGURE 11:**
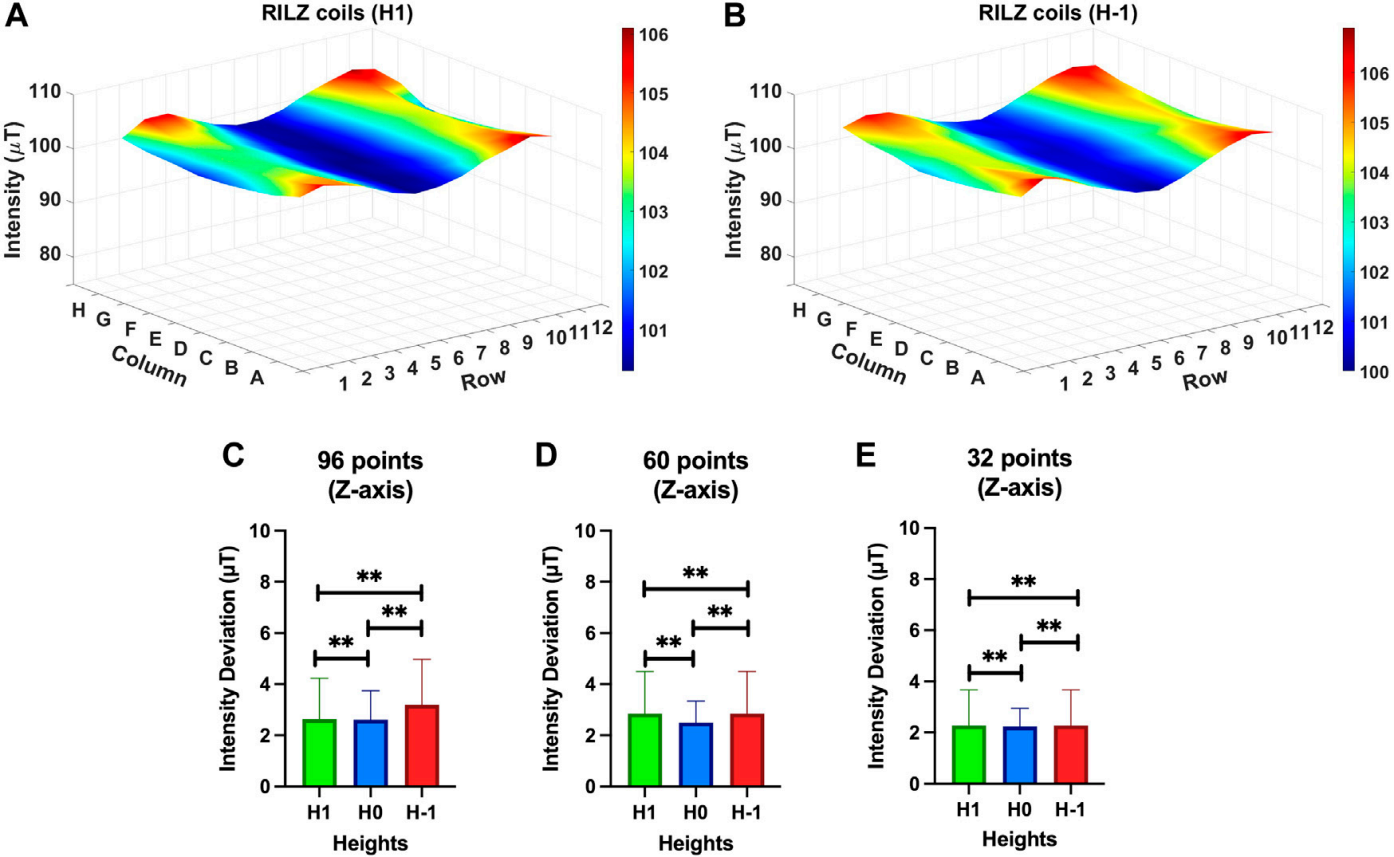
3D graphical representation of the intensity distribution of the measurements in the different heights H1 **(A)** and H-1 **(B)** of the RILZ coils system evaluated at 96 points, in the Z-axis when supplied with DC power with the centre point of the grid at 100 µT. Bar chart representing the mean and standard deviation of the deviations from the reference value of 100 µT on the Z-axis for the three heights evaluated, H1, H0, H-1, in the RILZ coil system for 96 points **(A)**, 60 points **(B)** and 32 points **(C)**. Spearman’s correlation statistic is applied with a 95% confidence interval. (**) *p*-value < 0.01.

Figures 11A, B shows the three-dimensional distribution of the intensity values obtained at heights H1 and H-1 with the RILZ coil system in the Cartesian Z axis, the main direction of application of the magnetic field. Qualitatively, it can be seen how both distributions are similar in shape and values, showing average deviations of less than 3.2 µT with respect to the reference value of 100 µT when evaluating the 96 measurement points, which corresponds to a difference of 0.5 µT with respect to the intensity deviation value obtained at height H0 (Figure 9E). When the number of wells decreases, so does the average intensity deviation, remaining at values of 1.8–3.2 µT regardless of the number of wells evaluated.

This similarity is corroborated through Spearman’s correlation statistic analysis (CI = 95%) between the deviation values above the reference value of 100 µT on the Z-axis between the three heights (H1 vs. H0; H0 vs. H-1, H1 vs. H-1) (Figures 11C–E). When evaluating 96 points, statistically significant correlation results are found when comparing H1 vs. H0 (Correlation index (C) = 0.896; *p* < 0.001), H0 vs. H-1 (C = 0.865; *p* < 0.001) and H1 vs. H-1 (C = 0.958; *p* < 0.001) heights. H0 (Correlation index (C) = 0.934; *p* < 0.001), H0 vs. H-1 (C = 0.914; *p* < 0.001) and H1 vs. H-1 (C = 0.922; *p* < 0.001). The same behaviour is observed when evaluating 32 points comparing the heights H1 vs. H0 (Correlation index (C) = 0.962; *p* < 0.001), H0 vs. H-1 (C = 0.965; *p* < 0.001) and H1 vs. H-1 (C = 0.948; *p* < 0.001).

These results combined with those shown in the 3-D graphs (Figures 11A, B) and the descriptive statistics results that can be consulted in Supplementary Table S2, allow us to ensure that there are no significant differences in the distribution of intensities between the different heights evaluated in the RILZ coil system.

### 3.3 Cellular results

In the following, the results obtained after performing the MTT cell test with both systems are presented. The main objective is to test whether the inhomogeneities in field intensity, which have already been reported in Section 3.2, have a direct effect on cell behaviour. Cells were exposed with both exposure systems simultaneously and the absorbance of the MTT signal was measured in three independent experiments.

**FIGURE 12:**
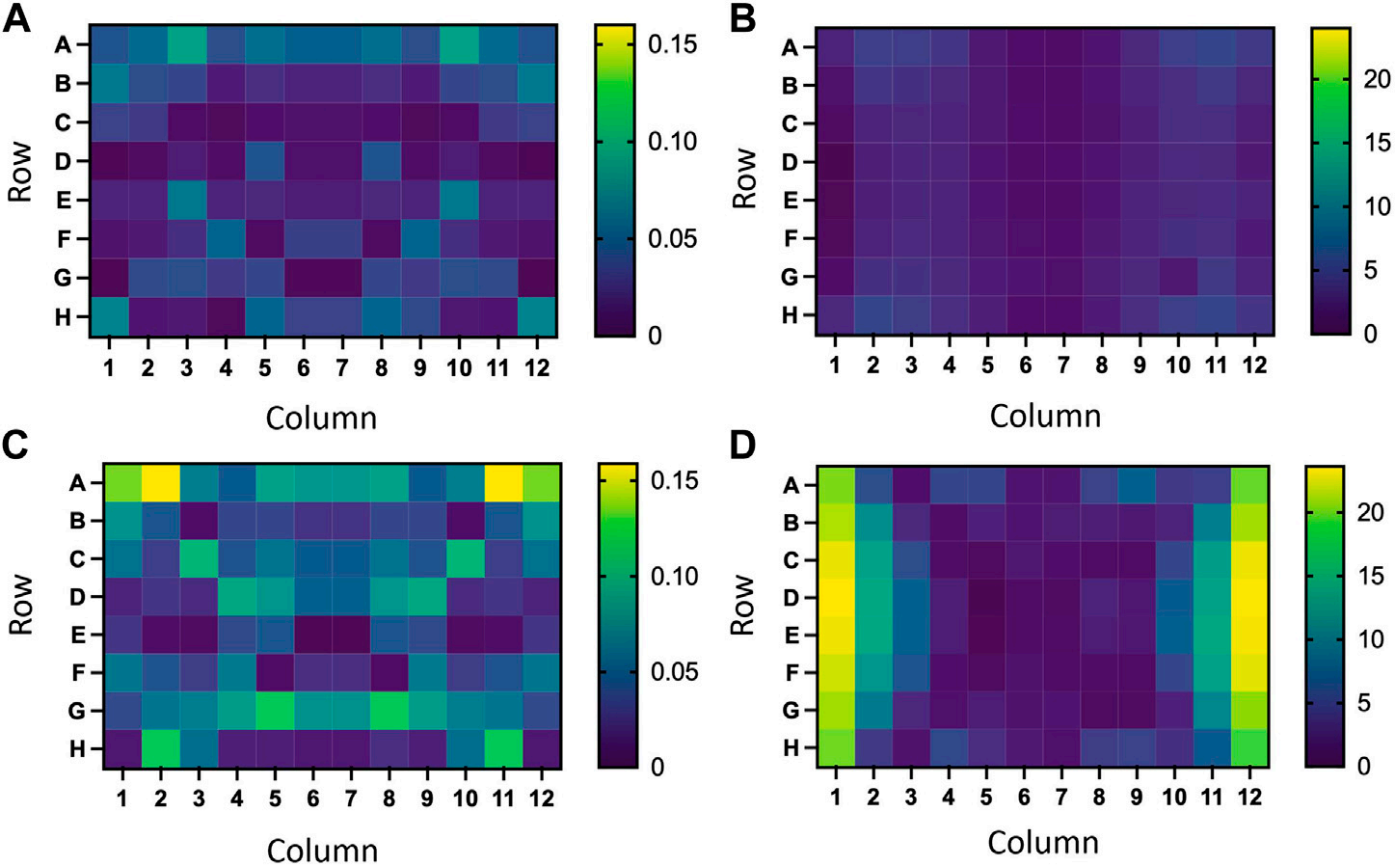
**(A,C)** Heat maps of the deviation distribution of the average absorbances in glioblastoma cells exposed to 50 Hz and 100 µT 72 h using a RILZ coil system **(A)** or a Helmholtz coil system **(C)** in a 96-well cell culture plate. B, **(D)** Heat maps of the deviation distribution of the average value of intensities in a 96-well cell culture plate with reference value 100 µT using a RILZ coil system **(B)** or a Helmholtz coil system **(D)**.

**FIGURE 13:**
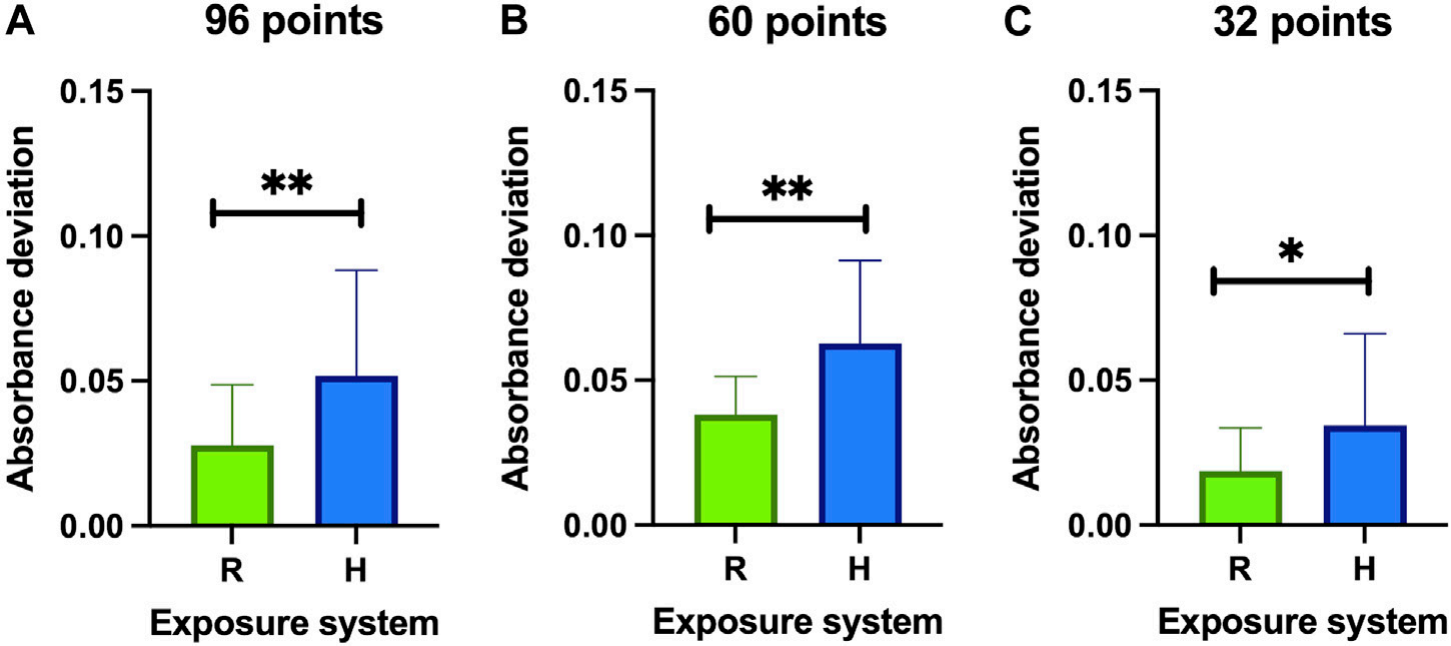
Bar graph representing the average ± SD of the absorbance deviation values from the average in three replicates exposed to a magnetic field of 100 μT and 50 Hz for 72 h with different exposure systems (R: RILZ, H: Helmholtz) in 96 points **(A)**, 60 points **(B)** and 32 points **(C)**. The ANOVA statistical test is applied with a confidence interval of 95%. (*) *p*-value<0.05; (**) *p*-value < 0.01.

As can be seen in Figure 12, the absorbance deviation values obtained with the RILZ system (Figure 12A) are less dispersed than those obtained with the Helmholtz system (Figure 12C). The average deviation value evaluated on all the points (96) of the plates exposed with the RILZ system (0.038 ± 0.013) is half that obtained with the Helmholtz exposure system (0.063 ± 0.029). The inhomogeneity of the absorbance deviation values can be compared with the inhomogeneities of the intensity distribution in Figure 12, where a larger difference between wells is detected in the intensity deviation values in the Helmholtz coil system (Figure 12D) *versus* the RILZ coil system (Figure 12B). This difference in the distribution of absorbance deviation values, as demonstrated in Figure 13, is statistically significant when evaluating 96 wells (*p* < 0.01) and 60 wells (*p* < 0.01) and, although statistical significance still exists at 32 wells (*p* < 0.05), it is lower, in line with the smaller difference in intensity distribution with the reduction in the number of spots evaluated between the different systems. Descriptive statistics related to the values of absorbance deviations can be found in Supplementary Table S3.

In summary, if these results are taken together with those already discussed of the distribution of magnetic field intensity values evaluated with measurements, it is determined that the homogeneous intensity values have a direct response on cell behaviour, returning more uniform responses in the absorbance data, which translates into a constant cell metabolic activity when the exposure is performed with the RILZ system.

## 4 Discussion

Because magnetic field magnitudes and directions depend on the location of the biological material (Misakian and Kaune, 1990), the measurement points must be sufficient to estimate the intensity variations throughout the volume of interest, and must be characterised for each axis (X, Y and Z). The cellular results obtained in this study suggest that small deviations of the magnetic field intensity value have direct effects on the behavioural response of biological systems. The maintained homogeneity in the distribution of intensity values in the RILZ system leads to a greater homogeneity in the cellular response when evaluating different sets of wells in a 96-well plate. In this study, the physical conditions of the exposure system (in this case the geometry which is the differentiating parameter), seem to be responsible for the observed uniformity in cell behaviour.

Although the differences in intensity distribution between the RILZ system and the Helmholtz system do not seem to be significant when 32 wells are evaluated, the cellular response obtained between the two systems does show significant changes, which could mean that cells are sensitive to very small variations in magnetic field homogeneity. In line with this hypothesis, several articles have been published showing differences in the behaviour of different cell lines exposed to very low magnetic field intensities, for example, changes in neurite outgrowth in PC12 differentiated with NGF (Blackman et al., 1993a; Blackman et al., 1993b; Trillo et al., 1996; McFarlane et al., 2000), changes in gene expression in myeloid leukaemia cells (Tokalov and Gutzeit, 2004) or in anti-tumour behaviour in carcinoma cells (Novikov et al., 2009) with magnetic fields at very low intensities. Most systems use the criterion of 5% or less magnetic field intensity variation to ensure homogeneity in the area of interest (Valberg, 1995); however, this value alone is not sufficient, as some systems validate homogeneity with magnetic field intensity values of 3600 μT at 30 Hz and present a variation of 200 μT, which corresponds to 4% deviation (Schuderer et al., 2004), other systems use magnetic field values of 10,000 μT at 50 Hz and present a variation of 300 μT, which corresponds to 3% deviation. Depending on the intensity value used in each of the above articles, the percentage of homogeneity used returns higher or lower values of accepted intensity deviation, not a fixed value at which effects on cell behaviour change are considered non-existent. The cellular results inevitably force us to carry out an experimental search for a minimum percentage of homogeneity that must be preserved when carrying out this type of study with replicas exposed to the same magnetic field that do not differ in intensity depending on the position they occupy in the same magnetic field generation system. This would allow replicable *in vitro* studies with optimal control of the magnetic field distribution whose results can be directly correlated to the exposure to the generated magnetic fields and not to the poor control over the exposure conditions.

The simulation comparison of the RILZ and Helmholtz coil systems in Figure 7; Table 1 provides an approximation of the vector behaviour (3 dimensions) of the magnetic field, where it is shown that the RILZ coils have a better intensity distribution with deviation values below 5 µT compared to the Helmholtz coil system. However, the simulation is only an approximation, and it is important to validate the system with real measurements to ensure the behaviour of the exposure system. Figures 9, 10, allows to delimit the area where the field homogeneity is guaranteed, having that in the RILZ coils the magnetic field is homogeneous in the 96 measurement points; being reduced in the Helmholtz coil to the 32 central points. While the simulation approximates the values of the magnetic field intensity, the intensity measurements show the real time values that occur in the three Cartesian directions. Once it has been determined that small variations in intensity give different responses in cell behaviour, it is necessary to mark the effective working area in which the field homogeneity is preserved with real values and not approximations.

In Figures 11, 12 the comparison between different heights is established to characterise the magnetic field distribution, obtaining that there are no significant differences between the heights, which allows the homogeneity area to be delimited in a volume determined by 3 96-well cell culture plates.

The main direction of application of the magnetic field is of great importance. The RILZ coil system presented here ensures that the main component of the magnetic field application is parallel to the cell culture surface in which the cells are immersed, with the other two spatial components having negligible magnetic flux values. Several studies have determined that there is a relationship between the direction of the applied magnetic field and the direction of cell growth in various cell lines such as stem cells (Sadri et al., 2017), bone cells (Lee and McLeod, 2000; Okada et al., 2021), glioblastoma (Ogiue-Ikeda and Ueno, 2004; Teodori et al., 2006) muscle cells (Umeno and Ueno, 2003; Ogiue-Ikeda and Ueno, 2004). Looking at the results found in the scientific literature, it could be assumed that cell behaviour differs depending on the main direction of application of the magnetic field.

The final purpose of bioelectromagnetic testing is to establish the mechanisms of interaction between biological systems and magnetic fields, and to this end it is necessary to produce magnetic fields in as controlled a manner as possible with the introduction of minimal external physical distortions. There are numerous communications reiterating the importance of controlling exposure parameters in the development of *in vitro* studies of exposure to magnetic fields (Misakian and Kaune, 1990; Misakian et al., 1993; Valberg, 1995; Misakian, 1997; Makinistian et al., 2018). As demonstrated in this study, small changes in field intensity modify cell behaviour, so failure to report pre-existing magnetic fields results in studies that cannot be replicated under different environmental conditions. One of the main problems *in vitro* bioelectromagnetic assays is the generation of contradictory results. As demonstrated in the study conducted, it is not possible to make comparisons of results using different exposure methodologies and which, in addition, do not take real measurements ensuring that the theoretical value of magnetic field intensity of the exposure is the real value.

The RILZ coil system described in this manuscript is suitable for *in vitro* controlled magnetic field exposure assays, ensuring field homogeneity that allows the use of a large cell culture volume, exposure mostly on a single Cartesian component and maintenance of this homogeneity at three different heights. Having a large field homogeneity area is of great importance in bioelectromagnetics *in vitro* assays, because it allows accurate statistics to be performed on several replicates subjected to the same conditions of exposure to magnetic fields from the same seeding. In addition, it facilitates the use of cell culture plates of different sizes limited to the homogeneity area of the coils without increasing the deviation of the intensity values at the extremes.

In this study, a magnetic field exposure system, RILZ configuration, has been developed with a geometrical configuration different from the conventional ones, which improves magnetic field intensity homogeneity in vitro studies compared to the traditional Helmholtz circular coil system. The results of the magnetic field intensity measurements and their statistical analysis reveal that the RILZ system achieves greater uniformity in the magnetic field intensity distribution in all Cartesian directions (x, y, z), which is essential to ensure reproducibility and consistency in cell experiments. Moreover, this homogeneity is preserved at three heights, making the RILZ system ideal for volume stimulation of cell cultures with low-frequency magnetic fields. The evaluation of cell metabolic activity shows that small inhomogeneities of the magnetic field intensity have a significant impact on cell behaviour. Cells exposed with the RILZ system show greater consistency and stability in their metabolic activity compared to cells exposed to the Helmholtz system, where significant differences in cellular response are observed due to the presence of inhomogeneous values of magnetic field intensity.

## Data Availability

The original contributions presented in the study are included in the article/Supplementary Material, further inquiries can be directed to the corresponding author.
