# Supplementary material for: Design and characterisation of a cell exposure system with high magnetic field homogeneity: RILZ coils
Source: Front Bioeng Biotechnol. 2024 Mar 8;12:1337899. doi: 10.3389/fbioe.2024.1337899 (PMC10957649; doi:10.3389/fbioe.2024.1337899)
Supplement: Supplementary file 1 [file DataSheet1.PDF]

Supplementary Material

Table 1. Descriptive statistics of the deviation values with respect to the reference value for the three Cartesian axes, X, Y and Z, of both exposure configurations, RILZ - HELMHOLTZ, obtained after taking real measurements for the mean height, h0, and the different well sections, 96, 60 and 32.

Coils system	Number of points	Deviation	Average (± SD) [μT]	Minimum value[μT]	Maximum value[μT]	Range (Maximum value - minimum value) [μT]
RILZ	96	D_x	0.52 (±0.43)	0.00	2.00	2.00
		D_y	1.33 (±0.96)	0.00	4.80	4.80
		D_z	2.61 (±1.13)	0.10	5.30	5.20
	60	D_x	0.51 (±0.42)	0.00	1.30	1.30
		D_y	1.10 (±0.74)	0.00	2.90	2.90
		D_z	2.50 (±0.84)	1.20	4.40	3.20
	32	D_x	0.54 (±0.45)	0.00	1.30	1.30
		D_y	1.13 (±0.75)	0.00	2.70	2.70
		D_z	2.24 (±0.71)	1.30	3.50	2.20
Helmholtz	96	D_x	2.86 (±0.79)	0.20	4.90	4.7
		D_y	5.31 (±5.71)	0.00	23.70	23.70
		D_z	7.18 (±7.49)	0.00	23.60	23.60
	60	D_x	2.94 (±0.64)	1.60	4.90	3.30
		D_y	3.20 (±3.03)	0.00	12.00	12.00
		D_z	4.39 (±4.52)	0.00	14.20	14.20
	32	D_x	3.05 (±0.62)	2.30	4.90	2.60
		D_y	1.92 (±1.42)	0.00	6.30	6.30
		D_z	2.55 (±2.35)	0.00	7.40	7.40

Table 2. Descriptive statistics of the deviation values with respect to the reference value for the three Cartesian axes, X, Y and Z, of both exposure configurations, RILZ - HELMHOLTZ obtained after taking real measurements for the heights h1 and h-1, and the different sections of wells, 96, 60 and 32.

Heigh	Number of points	Deviation	Average (± SD) [μT]	Minimum value[μT]	Maximum value[μT]	Range (Maximum value - minimum value) [μT]
H1	96	D_x	1.73 (±1.18)	0.00	5.30	5.30
		D_y	1.02 (±1.18)	0.00	5.90	5.90
		D_z	2.63 (±1.60)	0.30	6.10	5.80
	60	D_x	1.53 (±1.02)	0.00	3.50	3.50
		D_y	0.70 (±0.68)	0.00	3.30	3.30
		D_z	2.38 (±1.43)	0.30	4.90	4.60
	32	D_x	1.78 (±0.91)	0.10	3.50	3.40
		D_y	0.53 (±0.45)	0.00	1.80	1.80
		D_z	1.88 (±1.23)	0.30	3.90	3.60
H-1	96	D_x	1.14 (±0.75)	0.00	2.90	2.90
		D_y	1.04 (±0.86)	0.00	4.60	4.60
		D_z	3.19 (±1.77)	0.00	6.90	6.90
	60	D_x	0.89 (±0.56)	0.00	2.10	2.10
		D_y	0.89 (±0.65)	0.00	2.90	2.90
		D_z	2.85 (±1.65)	0.40	5.60	5.20
	32	D_x	0.94 (±0.59)	0.00	2.10	2.10
		D_y	0.83 (±0.56)	0.10	2.40	2.30
		D_z	2.28 (±1.40)	0.40	4.50	4.10

Table 3. Descriptive statistics of the deviation values from the mean of the absorbance values obtained when exposing glioblastoma cultures (CT2A) to 100µT, 50 Hz for 72 hours with both exposure systems.

Coils system	Average (± SD) [µT]	Minimum value[µT]	Maximum value[µT]
RILZ	0.03817±0.01319	0.01559	0.06717
Helmholtz	0.06251±0.02866	0.01837	0.12981
